# TIR/BB-loop mimetic AS-1 attenuates cardiac ischemia/reperfusion injury via a caveolae and caveolin-3-dependent mechanism

**DOI:** 10.1038/srep44638

**Published:** 2017-03-14

**Authors:** Yuanping Hu, Meiling Zhang, Xin Shen, Guoliang Dai, Danyang Ren, Linli Que, Tuanzhu Ha, Chuanfu Li, Yong Xu, Wenzheng Ju, Yuehua Li

**Affiliations:** 1 Key Laboratory of Cardiovascular Disease and Molecular Intervention, Department of Pathophysiology, Nanjing Medical University, 818 Tianyuan Dong Road, Nanjing 211166, Jiangsu, China; 2 Department of Pharmacy, The Binhu Hospital of Hefei, Hefei, Anhui, China; 3 Department of Pharmacy, The Binhu Hospital of Hefei, Hefei, Anhui, China; The First People Hospital of Hefei, Hefei, 230001, Anhui, China; 4 Affiliated Hospital of Nanjing University of Traditional Chinese Medicine, 155 Hanzhong Road, Nanjing, 210029, China; 5 Department of Surgery, East Tennessee State University, Campus Box 70575, Johnson City, TN 37614-0575, USA

## Abstract

AS-1, the TIR/BB loop mimetic, plays a protective role in cardiac ischemia/reperfusion (I/R) but the molecular mechanism remains unclear. The muscle specific caveolin3 (Cav-3) and the caveolae have been found to be critical for cardioprotection. This study aimed to evaluate our hypothesis that caveolae and Cav-3 are essential for AS-1-induced cardioprotection against myocardial I/R injury. To address these issues, we analyzed the involvement of Cav-3 in AS-1 mediated cardioprotection both *in vivo* and *in vitro*. We demonstrate that AS-1 administration significantly decreased infarct size, improved cardiac function after myocardial I/R and modulated membrane caveolae and Cav-3 expression in the myocardium. For *in vitro* studies, AS-1 treatment prevented Cav-3 re-distribution induced by H/R injury. In contrast, disruption of caveolae by MCD treatment or Cav-3 knockdown abolished the protection against H/R-induced myocytes injury by AS-1. Our findings reveal that AS-1 attenuates myocardial I/R injury through caveolae and Cav-3 dependent mechanism.

It is widely recognized that ischemic cardiomyocytes contribute to the activation of the innate immune response. During this process, the damaged cardiomyocytes release “danger” signals, which interact with pattern recognition receptors (PRRs) to activate the immune response. Multiple lines of evidence suggest that TLR/IL-1R MyD88-dependent signaling pathway serves to elevate NF-κB activity and plays an important role in mediating apoptosis and inflammation during myocardial I/R injury^1^,^2^,^3^. Our previous data have showed that inhibition of IL-1R/MyD88 interaction by a small-molecule mimetic AS-1 markedly attenuates myocardial I/R injury^4^. Specifically, we observed that administration of AS-1 to mice immediately before reperfusion following ischemia significantly protects the myocardium from I/R injury and attenuates I/R-induced production of inflammatory cytokines and the infiltration of neutrophils into the myocardium. Our observations suggest that the IL-1R-mediated MyD88-dependent signaling pathway contributes to myocardial I/R injury. A single dose (50 mg/kg) was used in the previous study. However, the pharmacokinetics and tissue distribution of AS-1 after injection await elucidation. So we aimed to investigate the pharmacokinetic (PK) parameters and the concentration of AS-1 in heart samples with single dose administration AS-1 in Wistar Rats in order to devise an adjusted bioequvalence assessment strategy for AS-1.

Recent studies have shown that caveolae and its coat protein, caveolin3 (Cav-3), play an important role in cardioprotection against I/R injury^5^,^6^,^7^. Caveolae are small (~100 nm in diameter), cholesterol- and sphingolipid- enriched flask-like invaginations of the plasma membrane. Caveolins act as scaffolding proteins, which are essential for caveolae formation. There are three caveolin isoforms: caveolin1 and caveolin2, which are expressed in multiple cell types, and muscle-specific isoform-caveolin3 (Cav-3). Cav-3 is the principal protein component of caveolae and can function as scaffolds interacting with a number of signaling molecules^8^,^9^. However, it is unclear whether caveolae and/or Cav-3 could be involved in AS-1-induced cardioprotection.

In the present study, we present new evidence that AS-1 could invoke cardioprotection via attenuating I/R-induced re-distribution of caveolae to the plasma membrane and by salvaging the loss of Cav-3 in cardiomyocytes.

## Results

### The chemic molecular parameters of AS-1 and the pharmacokinetics of AS-1 after injection to experimental rats

**Figure 1:**
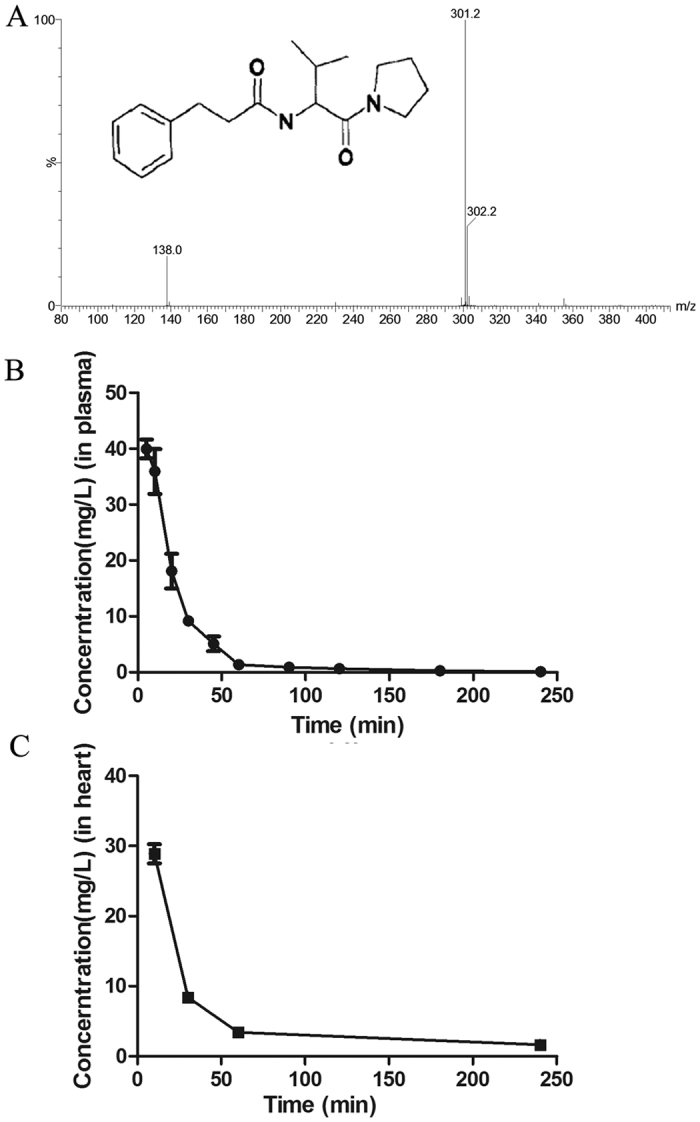
The structure and concentration-time manner of AS-1 in circulation. (**A**) Full-scan production spectra of [M–H]^−^ions and fragmentation pathways for AS-1; (**B**) Plasma concentration – time manner after interveinal injection 35 mg/kg in Wistar Rats (n = 4); (**C**) Heart concentration – time manner after interveinal injection 35 mg/kg in Wistar Rats (n = 4).

AS-1 was synthesized as described previously^10^ and the chemical structure of AS-1 was delineated (Fig. 1A). Then we designed a method of electrospray ionization with greater sensitivity and selectivity. In negative ion mode, AS-1, the molecular weight of which is 302, showed a peak at 301 m/z in methanol. We used a dosage of 35 mg/kg in rats, which was equivalent to the previously used dosage of 50 mg/kg in mice, to detect the partial main pharmacokinetic (PK) parameters of AS-1. The PK parameters of AS-1 after administration in Wistar rats (n = 4) are showed in Table 1. The maximum concentration (C_max_) and half life time (t_1/2_) of AS-1 after injection to rats were 39.94 ± 1.67 mg/L and 10.7 ± 0.79 min, respectively. As shown in Fig. 1B, the mean plasma concentration-time manner of AS-1 following injection declined very rapidly. Then, we determined the concentration-time curve in heart (Fig. 1C). We found AS-1 was present in heart and the clearance of AS-1 in heart was much slower than in plasma.

### AS-1 treatment reduces myocardial infarct size and improves cardiac function after I/R injury

**Figure 2:**
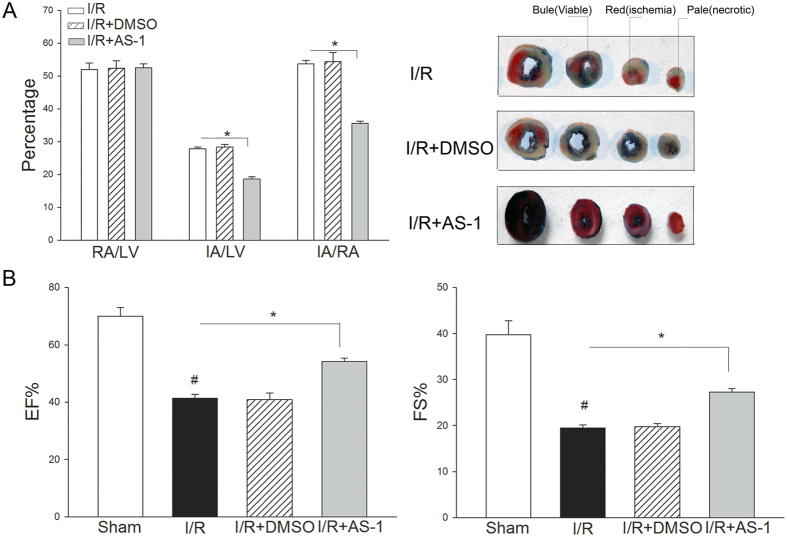
AS-1 decreased myocardial infarct size and improved cardiac function following myocardial ischemia/reperfusion injury. (**A**) Mice were treated with AS-1 or vehicle control, DMSO by i.p. injection immediately before reperfusion (4 h) after ischemia. Hearts were harvested and infarct size was determined by TTC staining. The infarct area (white) and the area at risk (red + white) from each section were measured using an image analyzer. Ratios of risk area vs. left ventricle area (RA/LV) and infarct area vs. risk area (IA/RA) were calculated and were presented in the graph. (**B**) AS-1 administration improved cardiac function following myocardial I/R. AS-1 or vehicle control, DMSO were injected immediately before reperfusion after ischemia (45 min). Cardiac function was examined 24 hours after I/R by echocardiography. Ejection fraction (EF%); fractional shortening (FS%). n = 6, ^#^P < 0.05 compare to sham; *P < 0.05 compared with indicated groups.

We next examined the therapeutic effect of AS-1 on myocardial infarct size following myocardial I/R injury. Mice were treated with AS-1 at the concentration of 50 mg/kg body weight immediately before reperfusion. After 45 min of ischemia and four hours of reperfusion, the hearts were harvested for the evaluation of infarct size. As shown in Fig. 2A, ischemia followed by reperfusion induced significant myocardial injury as denoted by the infarct size of mice. In contrast, AS-1 administration significantly decreased the ratio of infarct area/risk area (IA/RA) by 33.8% (35.5 ± 0.73% vs. 53.6 ± 1.15%, n = 6, P < 0.05) compared with untreated I/R group. Injection of vehicle control did not alter I/R-induced myocardial infarction. There was no significant difference in the ratio of risk area/left ventricle (RA/LV). We also examined the role of AS-1 administration in cardiac function following myocardial I/R injury. Cardiac function was assessed by echocardiography after reperfusion. As shown in Fig. 2B, transient myocardial ischemia followed by reperfusion significantly decreased ejection fraction (EF%) by 40.8% (69.9 ± 3.11% vs. 41.4% ± 1.24%, n = 6 mice/group, P < 0.05) and fractional shortening (FS%) by 50.9% (39.7 ± 3.08% vs. 19.5 ± 0.71%, n = 6 mice/group, P < 0.05) compared with sham group. In contrast, AS-1 administration attenuated I/R-induced suppression of cardiac function. The EF% and FS% values in AS-1 group were significantly increased by 40.5% (58.19 ± 1.13% vs. 41.41 ± 1.24%, n = 6 mice/group, P < 0.05) and 40.3% (27.30 ± 0.74% vs. 19.46 ± 0.71%, n = 6 mice/group, P < 0.05) respectively, when compared with the untreated I/R group. Treatment with the vehicle did not alter I/R-induced cardiac dysfunction.

### AS-1 treatment attenuates I/R induced the loss of membrane caveolae in the myocardium

**Figure 3:**
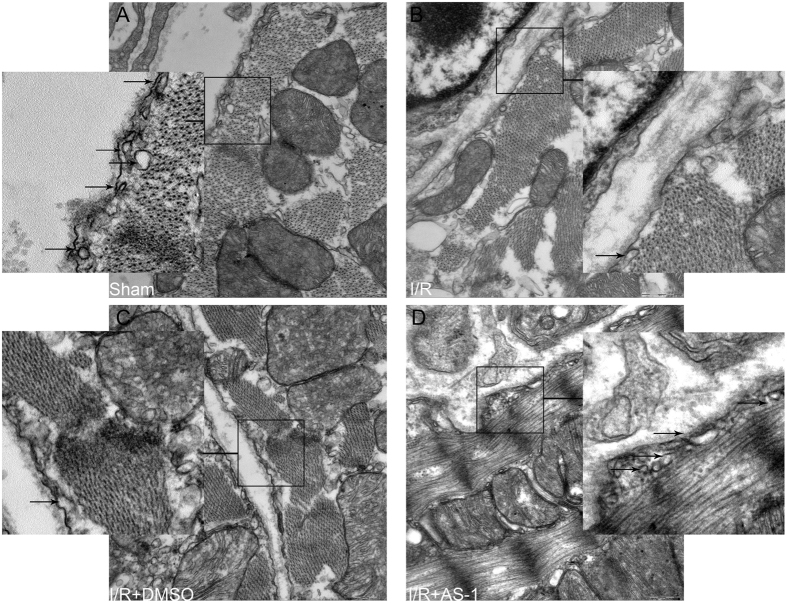
AS-1 modulated membrane caveolae after myocardial I/R injury. AS-1 was administered immediately before reperfusion after ischemia (45 min). Untreated I/R mice served as I/R control. Transmission electron microscopy showed I/R injury decreased the number of caveolae in the AAR of heart. AS-1 administration attenuated the decrease in the number of caveolae in heart compared with I/R group.

Caveolae are lipid rich micro-domains present as distinct molecular platforms for the regulation of cytoprotection^11^. Mounting evidence suggests caveolae can participate in ischemia, anesthetic, and opioid induced cardioprotection against I/R injury^5^,^6^,^12^. We examined the effect of AS-1 administration on membrane caveolae formation in the myocardium following myocardial I/R injury (Fig. 3). Transmission electron microscopy revealed that myocardial I/R injury reduced the number of membrane caveolae in the myocardium, when compared with sham control hearts. In AS-1 treated hearts, the numbers of caveolae were significantly increased compared with I/R control hearts, indicating that AS-1 treatment may attenuate I/R-induced loss of caveolae in the myocardium.

### AS-1 attenuates I/R induced a decrease in Cav-3 levels in buoyant membrane fractions (BFs)

**Figure 4:**
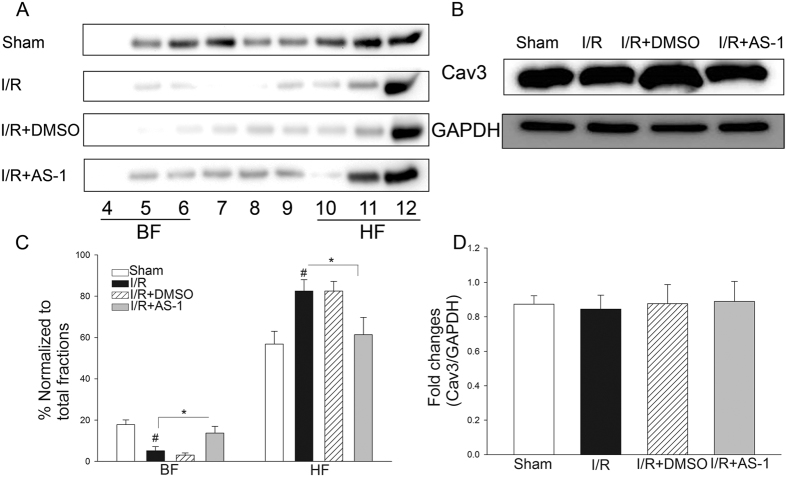
AS-1 attenuated I/R induced distribution of Cav-3 in the myocardium. The mice that were subjected to myocardial ischemia (45 min) were treated with AS-1 (n = 6) or vehicle control, DMSO, (n = 6) by i.p. immediately before reperfusion (4 h). Excised hearts underwent sucrose density fractionation. (**A** and **C**) AS-1 increased the enrichment of Cav-3 in BFs. Representative Western blots shows distribution of Cav-3. Cav-3 was decreased in BFs after I/R (representative immunoblots are shown), AS-1 attenuated the decrease of Cav-3 in BFs induced by I/R, and confirmed by densitometry normalized to total fraction amounts (**C**). Cav-3 densitometric results were combined into light fractions 4–6 and heavy fractions 9–12. (**B** and **D**) Hearts were harvested for the preparation of total proteins and the expression of Cav-3 was examined by Western blot. I/R injury or AS-1 treatment did not alter the expression of Cav-3 (**B**). n = 6, ^#^P < 0.05 compare to sham; *P < 0.05 compared with indicated group.

Cav-3 is a coat protein of caveolae. To find out the effect of AS-1 treatment on Cav-3, we examined Cav-3 levels in the myocardium with and without I/R injury. To this end, proteins isolated from heart tissues were fractionated using discontinuous sucrose gradient centrifugation. The fractioned proteins were analyzed by western blot with a specific Cav-3 antibody. As shown in Fig. 4A, I/R decreased Cav-3 levels in fractions 4 to 6 by 70.9% (17.78 ± 2.3% vs. 5.17 ± 1.9%, n = 6 mice/group, P < 0.05) compared with sham control. In contrast, AS-1 treatment partially blocked I/R-induced decreases in the levels of Cav-3 in the fractions of 4 to 6 by 1.7 fold (13.76 ± 3.2% vs. 5.17 ± 1.9%, n = 6 mice/group, P < 0.05). Figure 4B showed that neither I/R stimulation alone nor AS-1 treatment significantly altered the total Cav-3 levels in the myocardium. These results indicate that I/R injury may cause a re-distribution of Cav-3 from buoyant membrane fractions (BFs) to heavy fractions (HFs) without impacting overall Cav-3 levels and this process could be potentially blocked by AS-1 administration.

### AS-1 attenuates hypoxia/reoxygenation (H/R) induced cell death of cardiomyoblasts

**Figure 5:**
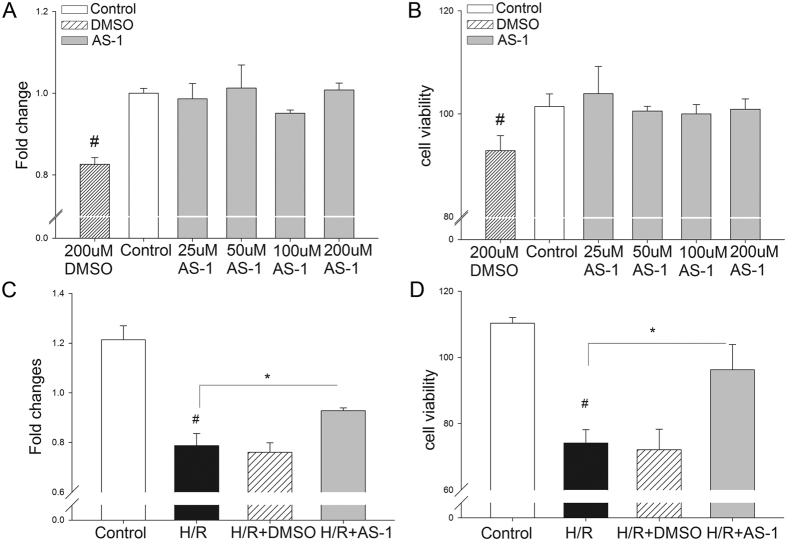
MTT and CCK8 assay on H9C2 cells. No toxicity of AS-1 were observed in H9C2 cells. H9C2 cells were treated with different concentrations of AS-1 (0–200 μmol/L). Cell injury was assessed by MTT assay (**A**) and CCK8 assay (**B**). AS-1 protects cells from H/R-induced injury. H9C2 cells were subjected to hypoxia for 1 hour followed by reoxygenation for 4 hours; AS-1 (50 μM) or DMSO were added into the cells before reoxygenation. The cells were subject to MTT assay (**C**) and CCK8 assay (**D**). n = 4, ^#^P < 0.05 compare to control; *P < 0.05 compared with indicated group.

To confirm that the effect of AS-1 on ischemic myocardium is not secondary to the influence of neurohumoral regulation, cardiomyocytes were subjected to hypoxia/reoxygenation (H/R). First, H9C2 cells were treated with or without AS-1 at the concentrations of 25–200 μM, which approximated the concentration of AS-1 in the heart. Cell viability was assessed by MTT assay and CCK8 assay. Figure 5A and B showed that AS-1 itself at the concentration of up to 200 μM did not influence cell viability, indicating that AS-1 used in the present study did not have toxic artifacts. Importantly, AS-1 (50 μM) added immediately before the cells were subjected to reoxygenation (4 h) after hypoxia (1 h) significantly attenuated H/R induced cell injury by 29.8% (96.3 ± 7.58% vs. 74.2 ± 4.00%, n = 4, P < 0.05) in MTT assay (Fig. 5C), and 18.3% (92.3 ± 1.1% vs. 78.7 ± 4.8%, n = 4, P < 0.05) in CCK8 assay (Fig. 5D), when compared to the DMSO control.

### AS-1 attenuates H/R-induced decreases of Cav-3 in buoyant membrane fractions (BFs)

**Figure 6:**
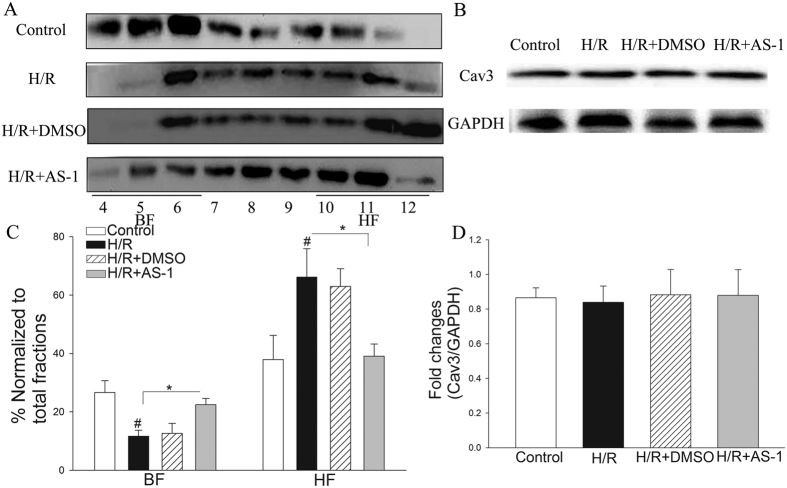
AS-1 attenuated the distribution of Cav-3 in neonatal cardiac myoctyes post H/R injury. Neonatal cardiac myocytes were treated with AS-1 (50 μM) or vehicle control, DMSO immediately before reoxygenation (4 h) after hypoxia in presence or absence of MCD (2 μM). Whole myocytes underwent sucrose density fractionation. (**A** and **C**) AS-1 increased the enrichment of Cav-3 in BFs. Representative Western blots depicting distribution of Cav-3. Cav-3 was decreased in BFs after H/R (representative immunoblots are shown), AS-1 attenuated the decrease of Cav-3 in BFs induced by H/R, and confirmed by densitometry normalized to total fraction amounts (**C**). Cav-3 densitometric results were combined into light fractions 4–6 and heavy fractions 9–12. (**B** and **D**) Whole cells were harvested for the preparation of total proteins and the expression of Cav-3 was examined by Western blot. H/R injury or AS-1 treatment did not alter the expression of Cav-3 (**B**). n = 4, ^#^P < 0.05 compare to control; *P < 0.05 compared with indicated groups.

Caveolar microdomains (enriched with cholesterol and located in BFs), together with caveolins, organize interactions between signaling molecules with caveolins thus playing a protective role during cardiac ischemia/reperfusion injury. To provide a solid cellular basis for AS-1 in the re-distribution of Cav-3 in the heart we isolated neonatal cardiac myocytes and subjected them to H/R injury. We treated myocytes with AS-1 at 50 μM or DMSO before the cells were subjected to reoxygenation (4 h) after hypoxia (1 h). After reoxygenation, the cells were harvested and cellular proteins were isolated and fractionated using a discontinuous sucrose gradient centrifugation. The fractioned proteins were subjected to immunoblot with a specific anti-Cav-3 antibody. Figure 6 showed that H/R significantly decreased the levels of Cav-3 in fractions 4 through 6 (BFs) by 56.2% (26.64 ± 4.02% vs. 11.68% ± 2.03%, n = 4, P < 0.05). In contrast, AS-1 treatment markedly attenuated H/R induced decreases in the levels of Cav-3 in fractions 4–6 by 101.0% (22.47 ± 2.17% vs. 11.68% ± 2.03%, n = 4, P < 0.05). Figure 6B showed that H/R stimulation or AS-1 treatment did not alter total Cav-3 expression in neonatal cardiac myocytes. These data indicated that AS-1 treatment may induce cellular redistribution of Cav-3.

### The protection of AS-1 was dependent on caveolae and Cav-3

**Figure 7:**
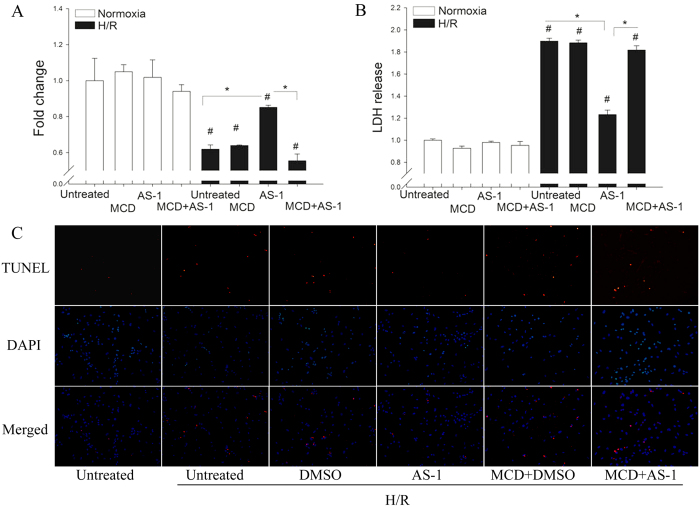
Disruption of caveolae abolished AS-1 induced protection against H/R-induced cell injury. H9C2 were treated with AS-1 (50 μM) before reoxygenation (4 h). Cell viability was assessed by MTT (**A**) and LDH release (**B**) in H9C2 treated with normoxia or H/R in presence or absence of MCD (2 μM). Normoxia untreated cells severed as control. n = 4, ^#^P < 0.05 compare to control; *P < 0.05 compared with indicated groups. Neonatal rat cardiomyoctyes were treated with AS-1 (50 μM) before reoxygenation (4 h). Representative pictures of TUNEL (red) and nuclear (DAPI, blue) staining (**C**) treated with normoxia or H/R in presence or absence of MCD (2 μM).

To examine whether AS-1 mediated protection against H/R-induced cell injury was through the caveolae dependent mechanism, we treated the cells with methyl-β-cyclodextrin (MCD) in order to disrupt caveolae^13^ before the cells were treated with AS-1. Figure 7A and B showed disruption of caveolae by MCD abolished AS-1-induced protection against H/R-induced injury in H9C2 cardiomyocytes: the levels of cell viability and LDH release in AS-1 plus MCD treated cells were comparable with H/R-stimulated cells.

To confirm the contribution of caveolae in AS-1 protection, we used TUNEL staining to detect cell apoptosis in neonatal rat cardiomyocytes post-H/R injury. Compared with untreated normoxic cells, TUNEL-positive cells were increased after H/R injury and AS-1 treatment reduced the number of apoptotic cells. However, the protection of AS-1 was abolished in cardiomyocytes that were pre-treated with MCD. These data suggest that caveolae are required for AS-1 induced cardioprotection.

**Figure 8:**
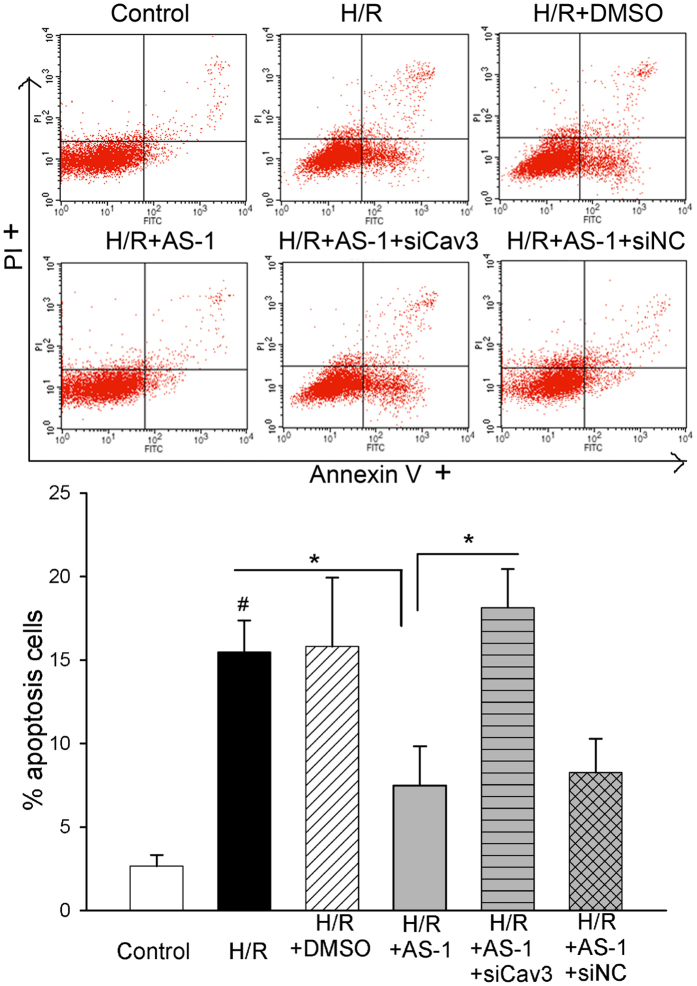
AS-1 protectes cardiomyocytes from H/R injury denpendent on Caveolin-3. Neonatal rat cardiomyoctyes were treated with AS-1 (50 μM) before reoxygenation (4 h). The cells treated with normoxia or H/R in presence or absence of MCD (2 μM) were stained by annexin V-FITC/propidium iodide (PI) and were analyed by flow cytometer. The number of apoptotic cells (annexin V positive, PI negetive) was presented in the graph. n = 5, ^#^P < 0.05 compare to control; *P < 0.05 compared with indicated groups.

To further extend the connections between Cav-3 and AS-1 effect on H/R injury, we tested whether protection promoted by AS-1 would depend, to some extent, on Cav-3. To this end, we used small inference RNA (siCav3) to knock down the expression of Cav-3. Transfection of siCav-3 reduced the level of Cav-3 by 76.7% compared with siNC group (data not shown). Then, we used PI and Annexin-V staining to detect cell apoptosis post H/R injury in cardiomyocytes. Figure 8 showed that the PI negative/Annexin-V positive cells were significantly increased post H/R injury, and AS-1 treatment suppressed cell apoptosis. Knocking down Cav-3 expression with siCav-3 before AS-1 administration abolished the suppression of AS-1 on apoptosis of cardiomyocytes induced by H/R injury. These data imply that Cav-3 is an important component for AS-1-mediated protection of cardiomyocytes from H/R injury.

## Discussion

The present study demonstrates for the first time that a TIR/BB-Loop mimetic AS-1 modulates caveolae and redistribution of Cav-3 in plasma membrane following I/R injury *in vivo* and *in vitro.* Specifically, we observed that AS-1 administration attenuated I/R-induced decreases in the amount of caveolae and BF-associated Cav-3 levels in the myocardium. Similar results were observed *in vitro* studies using cardiomyocytes subjected to H/R stimulation. Further, disruption caveolae by MCD or knocking down Cav-3 with siCav-3 completely abolished AS-1 mediated protection against H/R-induced cell injury. Our data suggest that the caveolae are essential for AS-1 induced protection against myocardial I/R injury.

We developed a sensitive, rapid, and selective LC-MS/MS method for analysis of pharmacokinetics of AS-1 in the blood after administrating it to rats. We observed that following i.v injection, AS-1 appeared in the blood quickly and then gradually decreased in the circulation. Tissue distribution showed that AS-1 was taken by the heart and located within the cells. This is the first report showing that AS-1 can be taken quickly by tissues.

We have previously reported that administration of a TIR/BB-Loop mimetic AS-1 immediately before reperfusion after ischemia protected the myocardium from I/R injury via suppression of NF-κB binding activity and inflammatory cytosine levels^4^. In the present study, we found that myocardial ischemia followed by reperfusion for 4 hours altered the morphology of the plasma membrane and decreased the number of caveolae in the myocardium. Moreover, I/R injury resulted in translocation of Cav-3 from BFs, one of the main components of cardiomyocytes caveolae, to HFs. Recent studies have shown that stresses can induce redistribution of Cav-3 from buoyant to heavy fractions without changing the overall expression levels of Cav-3 in cardiomyocytes and cardiomyoblasts^14^,^15^. These findings suggest that I/R injury couples the alteration of molecular signaling to changes in caveolae and Cav-3 distribution. We observed in the present study that administration of AS-1 immediately before reperfusion after ischemia significantly attenuated I/R-induced decreases in the numbers of caveolae in the plasma membrane and distribution of Cav-3. Caveolae and Cav-3 can anchor and regulate the function of proteins that modulate a variety of cellular processes^16^ and signal transduction^17^,^18^. In particular, Cav-3 plays an important role in ischemic preconditioning (IPC) induced cardiac protection in I/R injury^19^. We observed that disruption of caveolae by MCD and Cav-3 knockdown abolished the protection of AS-1 against H/R-induced cell injury, suggesting that loss of caveolae and/or re-distribution of Cav-3 in BFs could be an important mechanism for AS-1 induced protection against myocardial I/R injury.

In our previous study^4^, we reported that myocardial I/R markedly increased the association between IL-1R and myeloid differentiation primary response gene 88 (MyD88), which was associated with increased myocardial NF-κB nuclear translocation and binding activity, and enhanced the levels of inflammatory cytokines and adhesion molecules. We also reported that AS-1 administration attenuated pressure overload-induced increases in the levels of phospho-p38/p38 and phospho-ERK/ERK in the myocardium^20^. Interestingly, increased ERK activation in BFs during I/R was accompanied by a reduction in Cav-3 levels in these fractions. It is possible, therefore, that activation of the ERK isoforms in the light fractions may interact with Cav-3, resulting Cav-3 re-distribution and contributing to I/R injury^21^,^22^. Mechanistically, following H/R stimulation, MyD88 is recruited to TIR domain followed by formation of a complex with IRAK and TRAF6, leading to activation of MAPK family^23^,^24^. Activated MAPK can associate with Cav-3 to regulate downstream signaling, resulting in re-distribution of Cav-3 from BFs to HFs while simultaneously down-regulating the number of caveolae in plasm membrane. In the present study, we found that caveolae and Cav-3 are required for AS-1 induced protection. It is possible that AS-1 administration prevented I/R-induced association between IL-1R and MyD88, resulting in attenuation of p38 MAPK and ERK phosphorylation and redistribution of Cav-3 in BFs as well as the loss of caveolae from plasma membrane of cardiomyocytes.

We realized that there are some limitations in this study. Specifically, adult mice cardiomyocytes would have been a better choice for experiments instead of neonatal cardiomyocytes as used here. Moreover, a small-molecule compound that could reduce the expression of Cav-3 in BFs would be helpful to find out the role of Cav-3 redistribution on AS-1 cardioprotection. These pitfalls hopefully will be avoided in our future research.

In summary, we demonstrate that administration of AS-1 protects the myocardium from I/R injury and cardiomyocytes against H/R-induced injury. The mechanisms involve the remodeling of Cav-3-containing caveolae. Our data suggest that there is a link between IL-1R-mediated MyD88-dependent signaling and caveolae and Cav-3 in BFs.

## Materials and Methods

### Animals

Male C57BL/6 mice (7–8 weeks old) and Male Wistar Rats (250–280 g) were obtained from the Model Animal Research Center of Nanjing University (Nanjing, China). The animals were housed with free access to food and water. Experiments involving animals were conformed to the Guide for the Care and Use of Laboratory Animals published by the US National Institutes of Health (NIH Publication, 8^th^ Edition, 2011). All aspects of the animal care and experimental protocols were approved by the Nanjing Medical University Committee on Animal Care.

### myocardial I/R model in mice

The mouse model of myocardial I/R injury was induced by ligation of LAD as described previously^4^,^25^. Briefly, male mice were anesthetized with a mixture of ketamine (100 mg/kg i.p.) and xylazine (6 mg/kg i.p.) and ventilated. When adequacy of anesthesia was monitored by observation of slow breathing, loss of muscular tone, and no response to surgical manipulation, the hearts were exposed and the LAD coronary artery was ligated with a 6–0 silk ligature over a 1-mm polyethylene tube (PE-10). After completion of 45 min of occlusion, the coronary artery was reperfused, the thorax was closed, and the animals were extubated. Control male mice underwent a sham operation where the ligature around the LAD was not tied. Burprenox (0.36 mg/kg, i.m.) was employed as analgesia post-operative. AS-1 group mice were treated with AS-1 (50 mg/kg body weight) by intraperitoneal injection immediately before reperfusion after 45 min of ischemia. DMSO served as vehicle control.

### Infarct size measurement

Infarct size was determined by staining with triphenyltetrazolium chloride (TTC; Sigma-Aldrich) as described in our previous study^4^. Briefly, the hearts were removed and perfused with saline on a Langendorff system, followed by staining with 1% Evans blue (n = 6 mice/group). Each heart was sliced into 1-mm slices. The slices were counterstained with 1% TTC prepared with 200 mM Tris buffer (pH7.8) for 15 min at 37 °C. Viable nonischemic myocardium stains blue with Evans blue. Ischemic myocardium, which is still viable, stains red with TTC, whereas the necrotic myocardium does not stain and appears pale white. Images were analyzed by an image analyzer.

### Echocardiography

Echocardiography was performed with a two-D guide M-mode transthoracic echocardiographic examination (General Electric Co, Fairfield, Conn) as described in our previous study^4^,^25^. Mice (n = 6 in each group) were subjected to ischemia (45 min) followed by repercussion for 24 hrs. All measurements were made by one observer who was blinded with respect to the identity of the tracings. All data were collected from 10 cardiac cycles.

### LC-MS/MS conditions

Analyses were performed by an Alliance 2695 LC system (Waters, Milford, MA, USA) coupled with a triple-quadrupole tandem Quattro Micro mass spectrometer (Waters, Milford, MA, USA). Instrumental control is the Mass Lynx 4.1 software using for acquisition and processing of the data. The LC separation was performed on an Agilent Zorbax SB-C18 column (150 2.1 mm i.d. 5 mm, Agilent Technologies, Wilmington, DE, USA) by the mobile phase consisting of acetonitrile and deionized water (40:60, v/v) containing 10 mM at a flow rate of 0.8 mL/min with a security guard column (12.5 2.1 mm i.d. 5 mm, Agilent Zorbax SB-C18, DE, USA). The autosampler temperature was maintained at 15 °C. The total LC run time was 10 min with the column temperature kept at 30 °C.

The typical operating source condition for MS detector with an electrospray ionization (ESI) interface in negative ion mode. The detertion parameters were optimized as follows: capillary voltage, 2.7 kV; cone voltage, 45 V; source temperature, 110 °C; desolvation temperature, 350 °C; desolvation gas flow (nitrogen), 450 L/h; collision energy 28 V for limonin and 25 V for nimodipine; argon was used as the collision gas with the gas pressure of 3.0 × 10^−3 ^mbar.

### Sample preparation for pharmacokinetics of AS-1

Aliquots of 100 μL plasma and 10 μL of IS (internal standard, we use tinidazole as internal standard in this experiment) (0.967 mg/mL) were added into a 1.5 mL Eppendorf tube as previously described^26^. After vortex-mixing for 30 s, 500 μL ethyl acetate was added. Then vortex-mixed for 5 min. The organic phase was transferred to polypropylene tubes by centrifugation at 10,000 g for 10 min. After evaporation to dryness in the centrifugal thickener (Centrivap console, Labconco Co., USA) at 50 °C for 1 hr, the residue was reconstituted in 100 μL methanol and vortexed for 5 min, and then centrifuged for 10 min at 12,000 g. An 80 μL supernatant was injected onto the LC-MS/MS system for analysis.

### Pharmacokinetic examination of AS-1

The preclinical pharmacokinetic study of AS-1 was based on 4 healthy Wistar Rats. AS-1 at a dose of 35 mg/kg was injected to rats from tail vein. Blood samples of 400 μL were collected via a capillary in the venous sinus of orbit into heparinized tubes at 0, 5, 10, 20, 30, 45, 60, 90, 120, 180, and 240 min after single injection of AS-1. Plasma was collected by centrifugation for 10 min at 3,000 g and transferred to labeled plastic vials at 20 °C until analysis was carried out.

### Electron Microscopy

Whole hearts were fixed with 2.5% glutaraldehyde in 0.1 M cacodylate buffer overnight followed by post-fixed with 1% OsO_4_ in 0.1 M cacodylate buffer (1 h) and embedded as monolayers in LX-112 (Ladd Research, Williston, Vt) as described previously^7^. Sections were stained in uranyl acetate and lead citrate before observation with an electron microscope (JEOL 1200 EX-II, JEOL USA, Peabody, Mass; or Philips CM-10, Philips Electronic Instruments, Mahwah, NY). Caveolae were identified by their characteristic flask shape, size (50–100 nm), and location at or near the plasma membrane. Random sections were taken by an electron microscopy technician blinded to the treatments.

### *In vitro* experiments

H9C2 cells were purchased from Shanghai cellular institution and preserved and passaged by our laboratory. Neonatal rat ventricular myocytes were prepared from 1 to 2 day-old neonatal Sprague–Dawley rats as described previously^25^. The cells were incubated in Dulbecco’s Modified Eagle Medium (DMEM, Invitrogen Corporation, USA) supplemented with 10% (v/v) fetal bovine serum and 2 mmol/L L-glutamine, at 37 °C and 5% CO_2_ in humidified incubator. Bromodeoxyuridine (0.1 mM) was added into cultured neonatal rat ventricular myocytes 36 h after incubation prevent proliferation of cardiac fibroblasts. For induction of cellular hypoxia, cells were replaced by DMEM without glucose and incubated at 37 °C with 5% CO_2_, 1% O_2_ and 94% N_2_ in a hypoxia chamber (Thermo, HERA cell 150i) for 1 hour followed by incubation in normal incubator for 4 hours.

### Cytotoxicity assessments (MTT, CCK8 and LDH release assay)

MTT was dissolved in DMEM at the concentration of 5 g/L, filtered for sterilization. 25 μl of MTT solution or 10 ul CCK8 solution was added to each well containing cells (96-well microtiter plate). The plate was then incubated in a CO_2_ incubator at 37 °C for 4 hours and measured at 450 nm for CCK8 assay. For MTT assay, the media was removed, and 200 μl of dimethyl sulfoxide (DMSO) was instilled to each well, pipetted to dissolve crystals, and measured at 492 nm. The cells were divided into six groups; the concentration of AS-1 is 0 served as blank contrast, DMSO dissolvent group served as vehicle control. The absorbencies of different concentration of AS-1 solutions (25–200 μmol/L) were also measured. The LDH concentration in the culture medium was spectrophotometrically assayed using a kit from Pierce (catalog number 88954).

### Preparation of Sucrose Density Membrane Fractionation

Whole hearts were harvested, minced with a razor blade, homogenized for 30 s, and fractionated with sucrose density gradients as previous reported^21^. Twelve 1-mL fractions were collected, starting from the top of the gradient. Fractions 4 through 6 were buoyant membrane fractions (BFs) enriched in caveolins and proteins associated with caveolins. Fractions 9 through 12 were defined as heavy fractions (HFs). For Western blots, an equal amount of total protein from each fraction (10 μg) were analyzed.

### Immunoblot analysis

The cytoplasmic proteins (60 μg), whole heart lysate proteins (60 μg), the immunoprecipitation samples and fractions from sucrose density centrifugation were separated by SDS-PAGE and transferred onto polyvinylidene difluoride (PVDF) membranes (Amersham Biosciences) as described previously^25^. The membranes were incubated with primary antibodies (Cav-3 (Becton, Dickinson and Company) and GAPDH (Santa Cruz Biotechnology)) and followed by incubation with peroxidase-conjugated secondary antibodies. The signals were detected with the ECL system (Pierce). The signals were quantified by scanning densitometry with the Image J analysis system.

### Small interfering RNA (siRNA) transfection

To determine the role of Cav-3 in H/R injury, we developed siRNA to suppressing the expression of Cav-3, which was constructed and generated by Ribobio (Guangzhou, China). The target sense sequence: 5′-GCTACCTGATTGAGATCCA-3′. The siRNA transfection efficiency was confirmed by the Lipofectamine 3000 transfection commercial kit(Invitrogen, Shanghai, China). Neonatal rat ventricular myocytes were plated and after 36 hours cells were put on opti-MEM and subsequently transfected with siCav3 or siNC (negative control) for 4 h. Then opti-MEM was replaced by DMEM supplemented with 10% FBS. Subsequent experiments were carried out 48 h later.

### TUNEL Assay for Apoptosis

The TUNEL (terminal deoxynucleoitidyl transferase-mediated biotinylated UTP nick end labeling) assay was performed by *in situ* cell death detection kit (Roche, USA). The cardiomyocytes were washed by PBS three times and subsequently fixed for 60 min in 4% paraformaldehyde, pH 7.4, at room temperature. After being washed by PBS three times, the cardiomyocytes were permeabilized with 0.1% TritonX-100 in PBS for 10 min at room temperature. After being washed by PBS three tmes, apoptotic cells were detected by TUNEL staining following the manufacturer’s instructions. Finally, cardiomyocytes were counterstained with DAPI (Sigma, USA) for 5 min, at room temperature. Picture was taken in a blinded manner and the experiment was repeated for three times.

### Flow cytometer for apoptosis

Neonatal rat cardiomyocytes were washed three times with PBS, and lifted from the plates with 0.25% trypsin (invitrogen). Cells were washed with PBS three times, resuspended in PBS, and stained with 1 μg/mL PI (propidium iodide)(Sigma,USA) and annexin V (FITC) for 5 minutes, at room temperature, cells were analyzed on a flow cytometer. The PI and FITC-annexin-V integrals estimated the percentage of total cells that were alive (PI negative, annexin V negative) in early apoptosis (PI negative, annexin V positive) and necrosis (PI positive, annexin V negative)^27^.

### Statistical analysis

Data are presented as means ± SD. Comparisons between groups were performed using one-way ANOVA, and Tukey’s procedure for multiple range tests was performed. Value of P < 0.05 was considered to be significant.

## Additional Information

**How to cite this article**: Hu, Y. *et al*. TIR/BB-loop mimetic AS-1 attenuates cardiac ischemia/reperfusion injury via a caveolae and caveolin-3-dependent mechanism. *Sci. Rep.* **7**, 44638; doi: 10.1038/srep44638 (2017).

**Publisher's note:** Springer Nature remains neutral with regard to jurisdictional claims in published maps and institutional affiliations.

## Figures and Tables

**Table 1:** Pharmacokinetic parameters of AS-1 after interveinal injection of 35 mg/kg to Wistar Rats.

Parameter	Mean ± SD
C_max_ (mg/L)^a^	39.9434 ± 1.6862
AUC_0-tn_ (mg/L*min)^b^	1132.0412 ± 133.7931
AUC_0-∞_(mg/L*min)^c^	1143.0905 ± 112.5708
t_1/2_ (min)^d^	10.6843 ± 0.7853

^a^Maximum plasma concentration.

^b^The area under the plasma concentration time curve from 0 to tn.

^c^The area under the plasma concentration time curve from 0 to ∞.

^d^Half time. n = 4.
